# Advancements in Non-Addictive Analgesic Diterpenoid Alkaloid Lappaconitine: A Review

**DOI:** 10.3390/ijms25158255

**Published:** 2024-07-29

**Authors:** Wen Zhang, Shujuan Mi, Xinxin He, Jiajia Cui, Kangkang Zhi, Ji Zhang

**Affiliations:** 1 College of Life Science, Northwest Normal University, Lanzhou 730070, China; m212552562@163.com (S.M.); 18635662152@163.com (X.H.); cuijiajia@nwnu.edu.cn (J.C.); sqyjktwzkk@163.com (K.Z.); 2 Institute of New Rural Development, Northwest Normal University, Lanzhou 730070, China

**Keywords:** lappaconitine, alkaloids, analgesia, chemical modification, clinical application

## Abstract

The perennial herb *Aconitum sinomontanum* Nakai (Ranunculaceae) has been utilized as a traditional oriental medicine in China for numerous years. The principal pharmacological constituent of *A. sinomontanum*, lappaconitine (LA), exhibits analgesic, anti-inflammatory, anti-tumor, anti-arrhythmic, and anti-epileptic activities. Due to its potent efficacy and non-addictive nature, LA is widely utilized in the management of cancer pain and postoperative analgesia. This review encompasses the research advancements pertaining to LA including extraction methods, separation techniques, pharmacological properties, chemical modifications, and clinical applications. Additionally, it offers insights into the potential applications and current challenges associated with LA to facilitate future research endeavors.

## 1. Introduction

The genus *Aconitum* comprises approximately 300 perennial herbaceous species, exhibiting a wide distribution across Asia, Europe, and North America. In China alone, there are around 200 species distributed across various provinces, with a predominant presence in Yunnan, Sichuan, Tibet, Gansu, and other regions [1]. Among these species is *A. sinomontanum*, a traditional oriental medicinal plant widely utilized for treating diverse ailments including falls and injuries, joint pain, and gastroenteritis. Lappaconitine (LA) serves as the principal pharmacologically active constituent of *A. sinomontanum*, exhibiting a diverse range of pharmacological activities including analgesic [2], anti-inflammatory [3], anti-arrhythmic [4], antiepileptic [5], antitumor [6], and antioxidant effects [7]. Many countries (ex. India, Uzbekistan, Pakistan, Turkey, etc.) have used LA as a leading compound of plant extracts [8,9,10,11,12] and the utilization of LA hydrobromide as a non-addictive analgesic has been extensively practiced in clinical settings throughout China for numerous years [13]. Consequently, it has attracted numerous researchers to conduct comprehensive and systematic investigations on its properties.

This review comprehensively and meticulously elaborates on the progress related to LA from four aspects, including its extraction and separation, pharmacological activity, chemical modification, and clinical application (from 1990 to 2024). We believe that this review will assist researchers in gaining a better grasp of the current research status of LA and inspire further studies.

## 2. Extraction Method of Lappaconitine

LA, as an alkaloid with significant pharmacological activity, is mainly derived from the natural source of *A. sinomontanum*. However, with the deepening of research, LA was also successfully extracted from *A. leucostomum* [14] and *A. septentrionale* [15], a special product of Inner Mongolia, which further enriches the plant resources of LA.

The extraction methods of LA are primarily based on the general techniques for alkaloid extraction [16,17,18,19], including classical heating reflux extraction and cold immersion extraction. With the integration of modern technology in the field of traditional oriental medicine extraction and separation, ultrasound, microwave, and other advanced techniques have been introduced into the process of LA extraction, significantly enhancing its efficiency. Despite there being various methods available, they all share a common core principle: acid extraction and alkali precipitation.

### 2.1. Thermal Reflux Method

The fundamental principle of thermal reflux is to exploit the unique solvent properties for steam generation upon heating and reflow upon cooling. Through this characteristic, the solvent can cyclically move within the solid matrix, enabling rapid and effective dissolution and extraction of the target substance. This method significantly enhances mutual diffusion and migration between solid and liquid phases, thereby greatly improving extraction efficiency [20]. LA, a typical diterpenoid alkaloid, exhibits distinctive solubility properties. It readily dissolves in acidic solutions and acetone while also displaying good solubility in methanol and ethanol; however, it remains insoluble in solvents such as water or petroleum ether. Existing research reports often select ethanol and methanol as ideal extraction solvents for thermal reflux due to their excellent LA solubility [17,21,22]. The extraction efficiency of the thermal reflux method, however, is influenced by various factors, primarily including the solvent, extraction temperature, extraction time, and solid–liquid ratio. In order to comprehensively investigate the specific impact of these factors on extraction efficiency, extensive research has been conducted by scholars. In 2014, the thermal reflux method was employed by Xie et al. [16] to extract LA from *A. sinomontanum*, with ethanol serving as the solvent. By means of a combination of single-factor experiments and orthogonal experimental design, they systematically investigated the influence of various conditions on LA yield. The results demonstrated that the extraction temperature exerted the most significant impact, followed by solvent concentration, solid–liquid ratio, and extraction time. After conducting a series of experiments, they achieved the highest yield of LA at 0.80%. Subsequently, Zhang et al. [23] utilized methanol as their solvent, resulting in an LA yield of 0.81%.

### 2.2. Dipping Method

The dipping method, as a traditional extraction technique for natural products in traditional oriental medicine, can be categorized into cold dipping and hot dipping. The fundamental procedure involves placing the powdered traditional oriental medicine into an appropriate container, adding a suitable amount of solvent, and allowing the medicinal materials to soak for an extended period at room temperature or relatively mild conditions (60–80 °C), facilitating the dissolution of active components in the solvent [14,24]. The dipping method is widely employed in the extraction of natural active substances in traditional oriental medicine due to its straightforward operational procedure [24,25,26], particularly for extracting components that are heat-sensitive. However, it also exhibits evident drawbacks, namely relatively low extraction efficiency and prolonged extraction duration. Ethanol and benzene are commonly employed as solvents for the cold-leaching extraction of LA [14,25,26,27,28,29]. The cold-leaching extraction of LA from *A. sinomontanum* was conducted by Wei et al. [30] in 1980. Initially, the raw material was ground with sodium carbonate to liberate the alkaloid, followed by the addition of benzene for cold leaching extraction. After filtration, hydrochloric acid was introduced to the filtrate for agitation, subsequently alkalinized with ammonia water, and ultimately obtained through ether extraction. Upon concentration of the extract, LA was yielded at 0.50%. Subsequently, Zhang et al. [31] employed a similar approach to extract LA from *A. sinomontanum*, achieving a yield of 0.60%. Zhou et al. [32] extracted LA from *A. leucostomum* through cold soaking; 95% ethanol was utilized as the solvent and the extracts were obtained via multiple steps including cold leaching extraction, filtration, and concentration under reduced pressure. LA was successfully isolated with a yield of 0.60% by using column chromatography. Related studies also demonstrated [32,33,34] that LA constituted the most abundant component among alkaloids in *A. leucostomum*. Therefore, *A. leucostomum* holds potential as an innovative plant material for large-scale production of LA.

### 2.3. Ultrasound-Assisted Extraction

Although the heat reflux method and dipping method are widely employed for the extraction of bioactive substances, their inherent drawbacks cannot be disregarded, including the low extraction yield, high solvent consumption, elevated energy consumption, and prolonged duration [12,19,35]. What is even more concerning is that the presence of extraction solvent residue may pose a potential threat to human health. Therefore, it becomes particularly crucial to explore a more efficient and environmentally friendly extraction technology. Ultrasound-assisted extraction technology, with its energy-saving and highly efficient characteristics, has gradually garnered attention in the field of extracting bioactive substances [36]. Ultrasound can effectively disrupt the cellular membrane and enhance the solvent’s penetration into the target substance, thereby improving extraction efficiency. Sun et al. [37] employed three methods, namely ultrasound-assisted extraction, microwave-assisted extraction, and microwave-assisted ultrasonic extraction for extracting LA from *A. sinomontanum* and the extraction efficiencies of these three methods for LA were 0.887%, 1.208%, and 1.227%, respectively. The above research findings demonstrate that ultrasound-assisted extraction techniques exhibit superior efficiency in extracting LA compared to traditional solvent methods. This not only aids in reducing both solvent and energy consumption but also effectively shortens the extraction time and lowers production costs. Additionally, the utilization of a reduced amount of organic solvents during ultrasonic extraction mitigates potential health risks associated with residual solvent residues. However, due to its high energy consumption, ultrasonic-assisted extraction has yet to be implemented in the industrial production of LA.

### 2.4. Natural Eutectic Solvent Extraction

Natural eutectic solvents (NADES) are low eutectic mixtures consisting of two or more natural molecules blended in a specific molar ratio. The majority of these constituent molecules in NADES are derived from the primary metabolites of plants, including organic acids, sugars, polyols, and amino acids [38]. It is worth noting that the melting point of NaDES is lower than that of its individual components, which can be attributed to the presence of hydrogen bond interactions between the molecules [38]. Consequently, NADES exhibit remarkable stability in liquid form at room temperature. The ease of preparation and excellent biocompatibility offered by NADES, in comparison to conventional organic solvents, further enhance its potential for diverse applications across various fields, particularly in natural product extraction [39,40,41]. In the extraction process of natural products, the utilization of NADES as the extraction solvent not only enhances the efficiency of extraction but also serves as a sustainable and environmentally friendly solution. However, several parameters need to be optimized for achieving optimal extraction. These parameters encompass the solid–liquid ratio, extraction time, extraction temperature, and water content. Sharma et al. [42] employed 20 different NADES systems along with 4 traditional solvent systems to extract LA from *A. heterophyllum*. Their findings demonstrated that NADES (composed of lactic acid and glycerol mixed in a 1:1 ratio) exhibited the highest extraction efficiency of 7.99 ± 0.11 mg/g among all of the solvent systems tested. Subsequently, Sharma et al. further optimized the extraction parameters for the NADES system, resulting in a significant increase in LA yield from 7.99 ± 0.11 mg/g to 9.35 ± 0.28 mg/g under conditions including a solid–liquid ratio of 3:10. This finding provides novel methods and ideas for efficient and environmentally friendly LA extraction from plants.

## 3. Pharmacological Properties of Lappaconitine

LA exhibits a diverse range of potent pharmacological properties, primarily encompassing analgesic, anti-inflammatory, anti-tumor, anti-arrhythmic, and antiepileptic activities. Researchers have not only used a variety of pharmacological models to verify the activity of LA but also tried to preliminarily explore its mechanism of action. Hence, there are many reports on the pharmacological mechanism of LA; but, opinions are different.

### 3.1. Analgesic Activity

Compared to conventional analgesic drugs, LA exhibits distinct advantages. It not only possesses a broad spectrum of analgesic effects but also circumvents the common adverse reactions associated with opioids. Unlike opioids, LA does not induce severe side effects such as addiction and respiratory depression, making it particularly valuable in cases requiring long-term pain management and frequent medication [21,43]. Additionally, LA does not pose the risk of upper gastrointestinal diseases like non-steroidal anti-inflammatory drugs, further ensuring its safety [43]. LA possesses the property of preemptive analgesia, meaning it can exert its analgesic effects before the onset of pain, thereby providing patients with more timely pain relief. Furthermore, the analgesic effect of LA demonstrates a dose-dependent manner [44], enabling doctors to accurately adjust the dosage based on individual differences and pain severity in order to achieve optimal analgesic efficacy. It is worth mentioning that LA hydrobromide yields comparable analgesic effects to pethidine and is seven times more potent than aminopyrine, albeit with a slightly delayed onset time and prolonged duration of action [45]. The Randall–Selitto experiment also demonstrated that oral LA exhibits a significantly greater analgesic efficacy compared to oral morphine, thereby providing a novel option for moderate to severe pain management.

There are divergent perspectives within the academic community regarding the analgesic mechanism of LA. The prevailing consensus suggests that its analgesic efficacy is intricately linked to norepinephrine (NE), 5-hydroxy tryptamine (5-HT), P2X_3_ receptor, dynorphin A (DynA), and voltage-gated sodium channels (VGSCs or Navs) in a multifactorial manner. Ruan et al. [2] conducted an in-depth investigation into the impact of LA on P2X_3_ receptors of DRG neurons in CCI-induced neuropathic pain. LA exhibited analgesic properties by downregulating the expression of P2X_3_ receptor in DRG neurons, thereby elevating the pain threshold of CCI rats and diminishing the sensitivity and excitability of primary neurons. Xiao et al. [44] elucidated the analgesic mechanism of LA in the periaqueductal gray (PAG) area of rats by establishing a rat model of second-degree burn. The findings demonstrated that LA significantly alleviated burn-induced pain in rats by upregulating the expression and enhancing the functionality of P2X_3_ receptors in the PAG region and activating the endogenous analgesic system.

Subsequently, several studies have also demonstrated that analgesic targets for aconitine alkaloids, including LA, bulleyaconitine A, benzolyaconine, and bullatine A, are all localized in the spinal cord [46,47,48]. They all stimulate small keratinocytes in the spinal cord to release endogenous peptide dynorphin A and subsequently activate κ-opioid receptors on postsynaptic membrane neurons to induce analgesia. In 2018, the study conducted by Wang et al. [49] demonstrated that LA significantly enhanced the expression of target genes for prekinorphin and target proteins for dynorphin A in the spinal cord of rats with neuropathic pain, thereby eliciting analgesic effects.

However, it has also been reported that the analgesic mechanism of LA is associated with the descending inhibitory system of norepinephrine (NE) and serotonin (5-HT) [50,51]. The central analgesic effects of LA may be attributed to its ability to inhibit the reuptake of NE and 5-HT through presynaptic membranes, leading to an increase in NE levels within the synaptic cleft as well as the subsequent release of monoamine neurotransmitters that activate the descending inhibitory system. Nevertheless, studies conducted by Sun and Uieike Seitz [52,53] have demonstrated that the analgesic effect of LA in neuropathic pain models remains unaffected by either norepinephrine or the descending serotonin inhibitory system in the spinal cord. This finding contradicts previous reports and can be attributed to more intricate physiological conditions present in neuropathic pain models, where alterations occur in both the quantity and type of norepinephrine and serotonin receptors within the spinal cord, resulting in diverse physiological effects [54].

The voltage-gated sodium channel Na_v_1.7 is considered a primary target for the analgesic action of local anesthetics [55] and clinically-used drugs have been developed based on this mechanism [56,57]. It is believed that Na_v_1.7 is also involved in the analgesic mechanism of LA. In 2019, Gan’s research [58] unveiled that LA functions as an inhibitor for stably expressed Na_v_1.7 channels. In contrast to conventional local anesthetics such as tetracaine and bupivacaine, which promptly and reversibly inhibit Na_v_1.7 channels, LA demonstrates a gradual and irreversible inhibition potentially attributed to its molecular size; however, further validation is necessary to elucidate the specific reasons behind this phenomenon. Moreover, Sterling’s investigation [59] indicated that LA diminishes sodium influx while obstructing delayed potassium influx through binding with human heart cell sodium channel 2, thereby suppressing action potential generation in order to achieve its analgesic effect.

### 3.2. Anti-Inflammatory Activity

The persistent inflammation can induce profound cellular injury and excessive release of inflammatory mediators, thereby precipitating tissue and organ dysfunction [60,61]. Consequently, researchers have dedicated significant efforts to developing safer and more effective anti-inflammatory agents aimed at mitigating the severity of inflammation [62]. The efficacy of LA has been demonstrated in various inflammation models [63,64,65]. The anti-inflammatory mechanism of LA is believed to be closely associated with cyclooxygenase-2 (COX-2), inflammatory mediators, nuclear factor-κb, chemokines, cytokines, and free radicals [7,63,64,65,66,67].

Liu et al. [63] confirmed the anti-inflammatory effect of LA by utilizing inflammatory pain models, including formalin-induced inflammation, carrageenan-induced inflammation, and adjuvant arthritis in rats. Subsequently, Lei’s study also demonstrated that treatment with LA significantly reduced mechanical and thermal hyperalgesia in SD rats using a CFA (complete Freund’s adjuvant)-induced inflammatory pain model, while decreasing the transcription and expression of the OX-42 gene [64]. Huang et al. [65] further explored the anti-inflammatory effect of LA using a rat model of spinal cord injury and found that it effectively reduced cytokine expression (IL-1β, IL-10, and TNF-α), thus achieving an anti-inflammatory effect. The derivative of LA was observed to mitigate the inflammatory response by suppressing the production of NO, PGE2, and TNF-a through modulation of the Nf-κb and MAPK signaling pathways. Notably, LA’s derivative exhibited significant therapeutic effects on lipopolysaccharide-induced acute lung injury in vivo.

Shi et al. [7] conducted an investigation on the scavenging ability of its salt derivatives toward free radicals, which revealed that LA, along with its hydrobromide and hydrochloride forms, exhibit significant capabilities in scavenging free radicals and hold promising potential as natural antioxidants. Gao et al. [67] employed a molecular hybridization strategy to combine LA with classical nonsteroidal anti-inflammatory drugs (NSAIDs) in order to design novel lead compounds with enhanced anti-inflammatory activity. Among these compounds, derivatives of LA exhibited the most potent inhibitory effect on lipopolysaccharide (LPS)-induced NO production in RAW 264.7 cells while demonstrating minimal cytotoxicity. Molecular docking analysis revealed a binding affinity of -10.3 kcal/mol for the compound, indicating its ability to form a stable complex with cyclooxygenase-2 (COX-2), thereby effectively suppressing COX-2 activity. These findings suggest that the mechanism underlying the anti-inflammatory activity of LA may be associated with COX-2.

### 3.3. Anti-Tumor Activity

The current research has demonstrated the ability of LA to effectively inhibit various tumor cells [68,69]. However, due to its limited water solubility and bioavailability in clinical practice, the sulfate and hydrobromide forms of LA have predominantly been utilized to investigate its anti-tumor properties [68,70].

LA exhibits dose-dependent inhibition on the proliferation of A549 non-small cell lung cancer cells. Xu et al. [6] demonstrated that an increase in the concentration of LA resulted in a gradual accumulation of A549 cells in the G1 + G0 phase, accompanied by a progressive decrease in the populations of the S phase and G2 + M phase. Additionally, there was a gradual elevation in the apoptosis rate and a concomitant reduction in Cyclin E1 expression. Further findings by Ma et al. [71] revealed that LA LS significantly suppressed the expression of p-PI3K/PI3K, p-Akt/AKT, Cyclin D1, and Bcl-2 proteins (*p* < 0.05), while enhancing the expression of p53, p21, Bax, caspase 3, and caspase 9 proteins (*p* < 0.05). These results demonstrate that LA sulfate effectively arrests A549 cells at the G0/G1 phase through the PI3K/AKT signaling pathway to induce apoptosis and inhibit proliferation.

LA sulfate and LA hydrochloride have also been investigated for their anti-tumor effects on various cancer cell lines. LA sulfate has shown dose-dependent inhibitory effects on HepG2 and HeLa cells, with IC_50_ values of 360 μg/mL and 571 ± 0.42 μg/mL, respectively, after a 48-h treatment period [72,73]. LA sulfate induces apoptosis, arrests the cell cycle at the G0/G1 phase, and activates caspase pathways in hepatoma cells. In HeLa cells, its mode of action involves inhibiting Cyclin D1 and p-Rb proteins, promoting p21 and P53 protein expression, activating the mitochondrial-mediated Bcl2/Bax ratio and matrix metalloproteinases, and activating the Caspase9/7/3 pathway. Additionally, LA sulfate inhibits cellular proliferation through the PI3K/AKT/GSK3β signaling pathway. These findings suggest that LA sulfate has potential as a promising therapeutic agent for liver cancer treatment. LA hydrochloride has also demonstrated dose- and time-dependent inhibition of HCT-116 cell growth, with IC_50_ values of 413.1 μg/mL at 24 h and 174.2 μg/mL at 48 h [74]. Its inhibitory effects on tumor cell proliferation are attributed to its ability to arrest the cell cycle in the S phase and induce apoptosis through mitochondrial and MAPK signaling pathways, highlighting its potential as a promising treatment for colorectal cancer.

### 3.4. Anti-Arrhythmic Activity

Several studies have demonstrated the antiarrhythmic activity of LA in animal models. Wang et al. [75] showed its effectiveness in a mouse model in 1997 and Heubach et al. [76] reported similar findings in a guinea pig arrhythmia model, where preincubation with LA prevented aconitine-induced arrhythmia. Despite extensive research, the mechanism underlying the antiarrhythmic activity of LA remains inconclusive within the academic community. Kong et al. [77] effectively managed aconitine-induced arrhythmia in rats through the oral and intravenous administration of LA hydrobromide, which significantly increased the arrhythmia threshold. It is hypothesized that this therapeutic effect may be attributed to the modulation of calcium channels by LA hydrobromide.

Vakhitova et al. [78] investigated the molecular mechanism underlying the anti-arrhythmic effects of LA hydrobromide in an aconitine-induced arrhythmia rat model. The findings revealed that LA exerts its modulatory influence on genes involved in ionic current conductance formation at various stages of action potential (INa, Iks, IK1, and ICaT). Specifically, the expression levels of genes encoding Ca^2+^ channels (cacna1g), K^+^ channels (kcna6, kcnj1, kcnj4, kcnq2, and kcnq4), and vesicular acetylcholine transporter (slc18a3) were significantly upregulated in rats with arrhythmia following LA treatment. Conversely, mRNA levels of genes encoding K^+^ channels (kcne1 and kcns1), Na^+^ channels (scn8a), and membrane transporters (atp4a, slc6a9) exhibited a decrease. The SCN5A gene encodes Na_v_1.5, a prominent cardiac sodium channel implicated in various conductive arrhythmic diseases. The inhibition of Na_v_1.5 channels has been shown to effectively regulate arrhythmias [79,80,81]. Shults et al. [82] synthesized a series of LA derivatives based on LA and conducted molecular docking with the Na_v_1.5 channel protein, revealing significant binding activity and suggesting an inseparable association between LA and its anti-arrhythmic effects mediated by Na_v_1.5.

### 3.5. Anti-Epileptic Activity

The current body of research on LA’s role in treating epilepsy is limited; nevertheless, multiple studies have emphasized its potential as an antiepileptic agent. LA has demonstrated a selective reduction in neuronal epileptiform discharges and their burst duration induced by bicuculline [83], aconitine [84], or extracellular fluid lacking Mg^2+^ [85]. Additional investigations [5] suggest that LA can effectively suppress spike discharge during the later stages of epileptic seizures, thus exerting inhibitory effects against epilepsy. Ameri et al. [86] extensively explored the anti-epileptic mechanism of LA and found that it inhibited population spikes evoked by stimulation in stratum radiata and alveoli, along with field excitatory postsynaptic potentials (EPSP) recorded in stratum radiata CA1 for both normal and low Mg^2+^-treated rat hippocampal slices. The concentration of LA ranged from 3 to 100 mM, effectively suppressing the population spikes and field EPSP induced by the aforementioned stimuli. However, it is important to note that neuronal excitability was not affected by LA at concentrations below 100 mM and its effect did not increase even with higher stimulation frequency. The study conducted by Ameri [86] suggests that LA may have an antiepileptic role through inhibition of neuronal excitation; however, further investigation is required to elucidate its precise antiepileptic mechanism.

## 4. Modification of Lappaconitine Structure

Similar to other diterpenoid alkaloids, LA demonstrates significant toxicity. Consequently, researchers have focused their efforts on structural derivatization to mitigate its toxicity and enhance or broaden its pharmacological activities. Based on the available literature [64,67,82,87,88,89], the primary emphasis of LA’s structural derivatization has been on the benzene ring at position C-4, the two nitrogen atoms (20-N and 2’-N), and the easily derivatized hydroxyl groups. Specifically, scientists have obtained a variety of LA derivatives through molecular hybridization with other pharmacological groups or substitution of the aromatic ring of LA. Additionally, computer-aided drug design has been employed to explore structure–activity relationships [88]. The following section outlines two major strategies extensively reported for structural modifications of LA.

**Figure 1:**
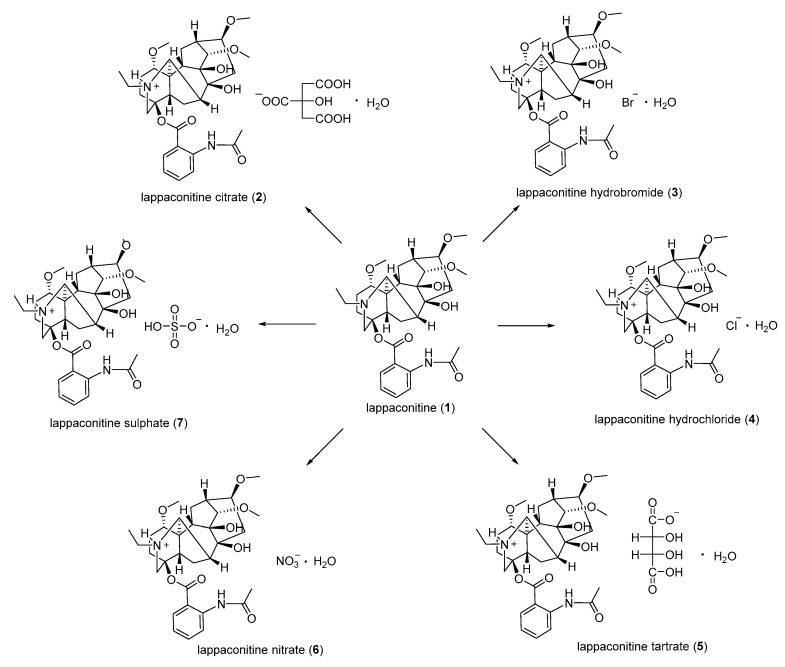
(Adapted from Sun et al. [70]). Synthetic strategy and lappaconitine derivatives (**2**–**7**).

Sun et al. [70] synthesized a series of LA salts **2–7** by using LA as the starting material and reacting it with corresponding organic or inorganic acids (Figure 1); they then assessed their analgesic activity through the hot plate method. The experimental findings indicate that at equivalent dosages, LA citrate, LA sulfate, and LA hydrochloride all exhibit superior onset times compared to the positive control (LA hydrobromide). Moreover, LA sulfate **7** demonstrates a more favorable log *p* value and exerts the strongest analgesic effect.

**Figure 2:**
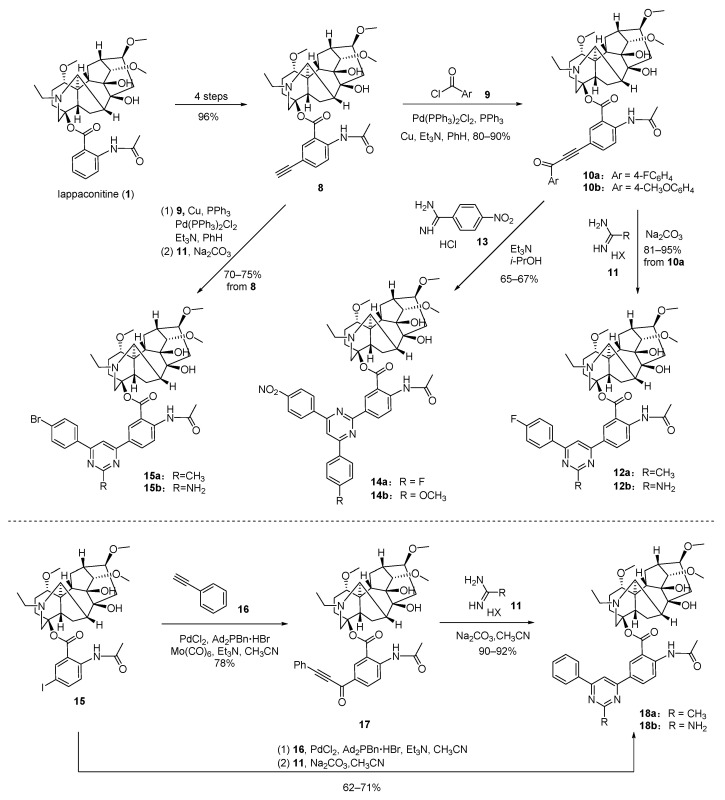
(Adapted from Shults et al. [88]). Synthetic strategy and lappaconitine derivatives (**12**, **14**, **15** and **18**).

Feng et al. [87] synthesized a series of acetal compounds derived from LA by utilizing LA and aromatic aldehydes as starting materials (Figure 2), followed by evaluating their anti-tumor activity. The synthesized compounds were subjected to an MTT assay to assess its inhibitory effect on the proliferation of tumor cells including HepG2, A-549, and HCT-8. LA and the marketed anti-tumor drug 5-fluorouracil were used as positive controls. LA and most of its derivatives exhibited significant inhibition against the proliferation of all three types of tumor cells, with the derivative bearing a 3-hydroxy-4-methoxybenzaldehyde showing particularly promising results. The IC_50_ values for HepG2, A549, and HeLa tumor cells were found to be 4.86 μM, 6.45 μM, and 3.29 μM, respectively, comparable to the inhibitory effect observed with the control drug 5-FU but higher than that achieved with LA alone. Further analysis revealed that the incorporation of vanillin groups into LA derivatives enhanced their solubility and permeability without altering the pharmacophore or dominant structure of LA itself, thereby augmenting their anti-tumor activity.

Shults et al. [88] reported a convenient approach to constructing heterozygous molecules containing diterpenoid alkaloid LA and a pyrimidine motif, along with an investigation into the antinociceptive potency of new compounds (Figure 2). Firstly, LA is utilized as a starting material to synthesize 5’-ethynyllappaconitine **8** through a previously established substitution method consisting of four steps. Then, the LA ynones **10a** and **10b** are obtained by employing transition-metal-catalyzed cross-coupling reactions between **8** and aroyl chlorides **9** *via* Sonogashira cross-coupling. Subsequently, efficient conversion of compounds **10a** and **10b** into 2,4,6-trisubstituted pyrimidines (**12a**, **12b**, **14a** and **14b**) is achieved by refluxing with acetamidine hydrochloride **11** or benzamidine hydrochloride **13** under alkaline conditions. Afterward, the authors optimized the two-step Pd-catalyzed Sonogashira coupling and condensation by integrating them into a one-pot procedure, resulting in pyrimidines **15a** and **15b** with yields of 70% and 75%, respectively, utilizing the aforementioned reaction system. The practical Beller's ligand, PdCl_2_, and versatile CO source-molybdenum hexacarbonyl Mo(CO)_6_ were employed in the three-component reaction of 5′-iodolappaconitine **15** with phenyl acetylene **16**, resulting in the formation of 5′-(1-oxo-3-phenylprop-2-in-1-yl)lappaconitine **17**. Then, the condensation of alkynone 19 with 13 led to the desired pyrimidines: **18a** and **18b**. This reaction sequence can also be smoothly carried out in a one-pot procedure. The analgesic activity was assessed using the acetic acid writhing assay and hot plate assay. Both oral and intraperitoneal administration of doses at 5 and 1 mg/kg were employed for the hot plate and acetic acid-induced writhing assays. Compounds **14a**, **14b**, and **15a** exhibited comparable activity to diclofenac sodium (10 mg/kg) and high-dose LA (5 mg/kg), which are standard drugs in this context. Notably, compound 16a demonstrated significant analgesic effects in both pain tests, potentially attributed to its substitution with a fluorophenyl group at position 2 of the pyrimidine ring. Additionally, compound **14a** displayed moderate oral toxicity (LD_50_ value greater than 600 mg/kg), which is 20 times lower than that of the parent compound LA; it also exhibited lower cytotoxicity in vitro experiments.

**Figure 3:**
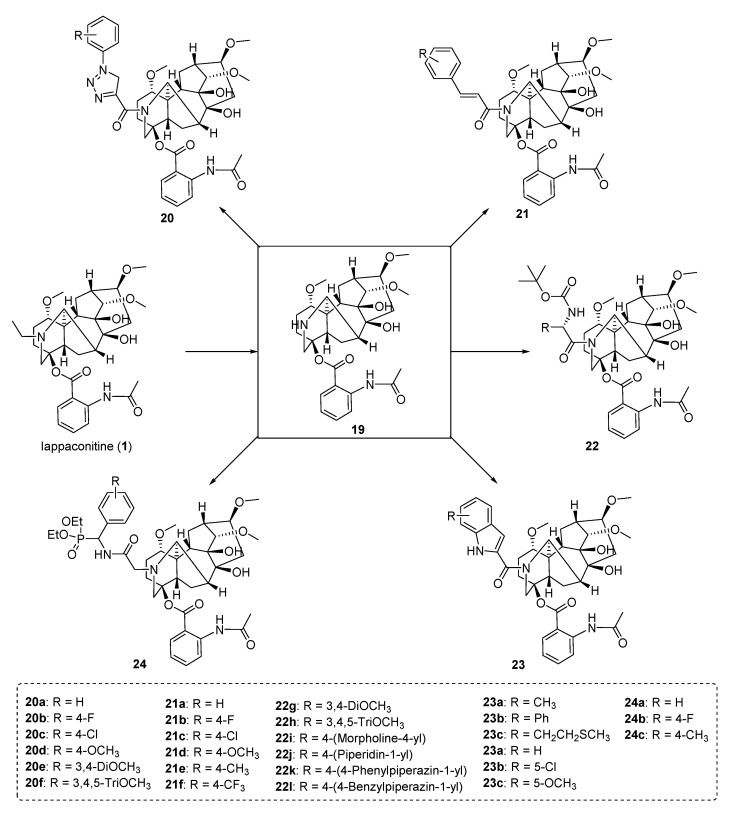
(Adapted from Quan et al. [66]). Synthetic strategy and lappaconitine derivatives (**20**–**24**).

Quan et al. [66] selected LA as the lead compound and synthesized 27 derivatives of LA (Figure 3), which were subsequently evaluated for their cytotoxicity and anti-inflammatory activity. Initially, *N*(20)-diethyllappaconitine **19** was prepared by reacting LA with NBS in an acetic acid solution. Compound **19** was then reacted with various natural product derivatives using EDC and HOBt as condensing agents to obtain target compounds **20–23**. Additionally, a *N*-hydrocarbonation reaction of compound **19** with chlorophospholipid derivatives yielded different types of target compounds **24**. The novel LA derivatives were assessed for their cytotoxicity in mouse RAW 264.7 macrophages while investigating the potential correlation between anti-inflammatory activity and cell viability. Compared to LA, all derivatives except compound **24b** exhibited excellent inhibitory ability, particularly compound **20d**, which demonstrated the most effective inhibitory effect with an IC_50_ value of 12.91 mmol/L.

**Figure 4:**
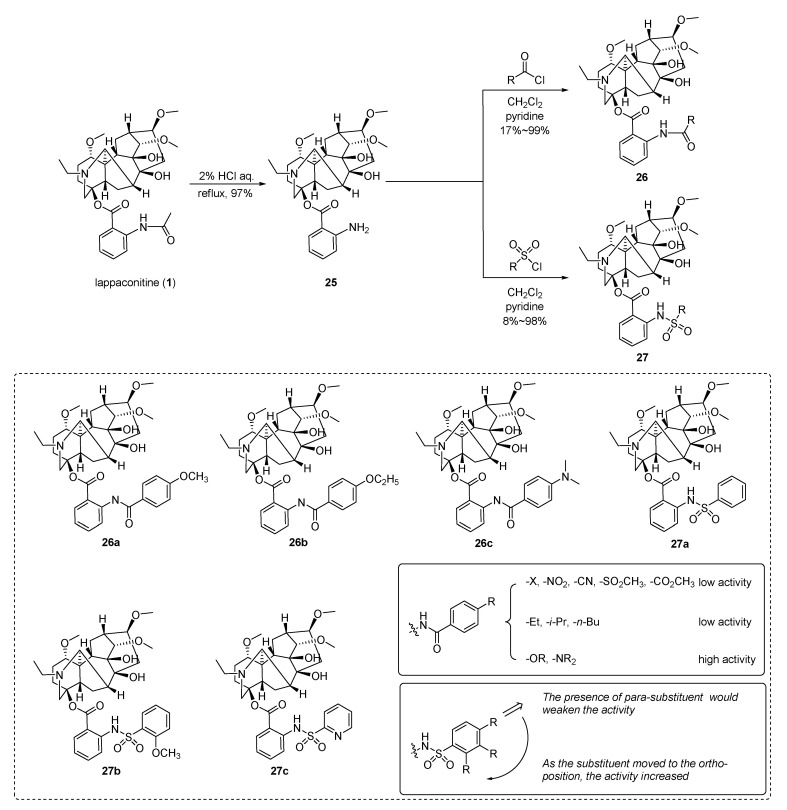
(Adapted from Zhou et al. [89]). Synthetic strategy and lappaconitine derivatives (**26**, **27**).

Zhou et al. [89] designed and synthesized two series comprising a total of 93 derivatives of LA through the modification of the C4 acetylaminobenzoate side chains. Then, the in vivo analgesic activity and toxicity of the compounds were evaluated and their structure–activity relationships were summarized. The two series of derivatives of LA are divided into amide and sulfonamide groups and the modification route is shown in Figure 4. Under acidic conditions, LA is hydrolyzed to *N*-deacetylated LA **25** with a yield of 97%. Subsequently, the corresponding compound is obtained by amidation or sulfonation with the corresponding acyl chloride or sulfonyl chloride with a yield of 17–99% and 8–98%, respectively. The data of the acetic acid mouse twisting model showed that six lead compounds (**26a**, **26b**, **26c**, **27a**, **27b**, and **27c**) had similar analgesic activity compared to LA (inhibition rates of all six derivatives were >60%) but that their toxicity was significantly reduced (ED_50_ and LD_50_ of the six derivatives were higher than LA) and that the therapeutic index TI of these compounds was 14–30 times that of LA. Based on this, the structure–activity relationship of LA amide and sulfonamide derivatives suggests that in phenyl-substituted amide derivatives, the inhibitory effect is attenuated when the substituent is positioned ortho or meta to the phenyl group, while para substitution exhibits superior activity, particularly with electron-donating groups in the para position (such as alkoxy or *N*,*N*-dimethylamino), which enhances the inhibitory effect. In phenyl-substituted sulfonamide derivatives, the position of the substituent exerts a more significant influence on the inhibition rate compared to amide derivatives (ortho > meta > para). Enhanced activity is observed when the substituent is located at ortho.

**Figure 5:**
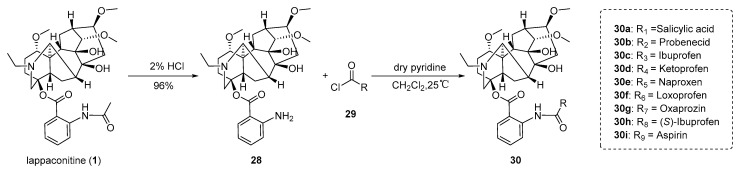
(Adapted from Shan et al. [67]). Synthetic strategy and lappaconitine derivatives (**30**).

Shan et al. [67] utilized LA as the initial substrate and employed a molecular hybridization strategy to synthesize a series of LA derivatives through hybridization with various NSAIDs (Figure 5). The anti-inflammatory activity of these derivatives was evaluated using an in vitro lipopolysaccharide-induced RAW 264.7 cell NO inflammation model. The precursor **28** was obtained through acid hydrolysis of LA with a yield of 96%. It successfully reacted with the corresponding acyl chlorides of NSAIDs **29a–29i**, which were generated from various NSAIDs (including Aspirin, Probenecid, Ibuprofen, Ketoprofen, Naproxen, Loxoprofen, Oxaprozin, and (*S*)-(+)-Ibuprofen) as well as salicylic acid. The reaction took place in dry pyridine and dichloromethane at a temperature of 25 °C. Consequently, the target compounds **30a–30i** were obtained with yields ranging from 40% to 57%. The synthesized compounds **30a–30i** exhibited a higher inhibition rate of NO generation compared to LA (16.8 ± 2.3%), indicating significant anti-inflammatory activity. Among them, compound **30e** exhibited a four-fold increase in the inhibition rate of NO generation, reaching 62.5 ± 1.3%, thereby suggesting that the presence of the naphthalene ring in NSAID contributes to enhancing its anti-inflammatory activity. The in vivo carrageenan-induced hind foot swelling model in rats demonstrated that compound **30e** displayed superior anti-inflammatory activity compared to LA and naproxen. To gain deeper insights into the interaction between compound **30e** and COX-2, molecular docking results revealed a strong coordination effect between compound **30e** and the active site of COX-2, forming a stable complex between the two.

**Figure 6:**
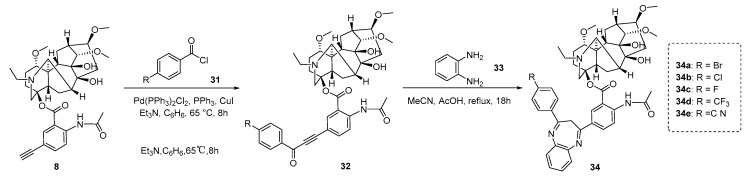
(Adapted from Shults et al. [82]). Synthetic strategy and lappaconitine derivatives (**34**).

In the same year, Shults et al. [82] proposed a straightforward approach for the synthesis of lappaconitine-1,5-benzodiazepine derivatives and evaluated their analgesic activity using the acetic acid writhing test and hot plate method. 5’-ethynyllappaconitine **8** underwent a sonoashira reaction with benzoyl chloride **31a–31e** to obtain lappaconitine alkynyl ketones **32a–32e**. The cyclic condensation reaction with *o*-phenylenediamine 33 was then conducted to obtain lappaconitine-1,5-benzodiazepine **34a–34e** with a yield ranging from 66% to 76%. Figure 6 presents the analgesic activity data of LA and its five derivatives, namely lappaconitine-1,5-benzodiazepines **34a–34e**, in an acetic acid-induced torsion test and hot plate test (oral) at effective doses of 5 mg/kg. Diclofenac sodium was used as the comparative drug at an effective dose of 10 mg/kg. Experimental results revealed that compound **34a** exhibited significant analgesic activity in both analgesic models among the five derivatives. It is noteworthy that the analgesic activity of derivative **34a** of LA at a dose of 5 mg/kg is comparable to that of diclofenac sodium at a dose of 10 mg/kg, further substantiating its potential as an analgesic agent. Compound **34a** exhibited significant analgesic effects in both pain tests, which may be attributed to the substituent properties at position 4 of the benzodiazepine ring. Meanwhile, following the Kerber method, acute toxicity tests were conducted on CD-1 mice using a single gavage administration of compound **34a**, which demonstrated moderate toxicity with an LD_50_ > 1500 mg/kg (oral). Importantly, it was observed that its toxicity was approximately 50 times lower than that of reference LA (with an LD_50_ range of 20–32.4 mg/kg). This modified approach, as a result, holds significant potential for enhancing the analgesic activity of LA while simultaneously mitigating its toxic effects.

## 5. Clinical Application of Lappaconitine

The clinical application of LA as a non-addictive analgesic has been extensively established in China for numerous years, particularly in the management of cancer pain and postoperative pain relief. Additionally, certain researchers have also explored its potential as a supplementary anti-inflammatory agent to address inflammation or anesthesia-related concerns.

### 5.1. Analgesia for Cancer

In comparison to traditional opioid analgesics, LA demonstrates characteristics such as low addiction potential, minimal occurrence of side effects, and an excellent safety profile. Over the past decades, several researchers [90,91,92,93,94,95] have investigated the efficacy of combining LA with other analgesics to alleviate cancer pain and have achieved satisfactory results.

Chen et al. [90] investigated the use of LA monotherapy for managing cancer-related pain. LA hydrobromide was compared to pethidine for pain management in liver cancer patients. While no significant difference was observed after one week, the experimental group receiving LA hydrobromide showed a significantly higher effective rate (92.35%) compared to the control group (18.18%) after three weeks. This indicated that LA had a longer duration of action, providing a stable curative effect and better patient compliance.

Several studies have also confirmed that the combination therapy of LA and various drugs for cancer pain not only exhibits good analgesic effects but also improves patient dependence and significantly reduces adverse reactions. The combination of fentanyl and LA was investigated by Chen et al. [91] for treating patients with advanced cancer. Although there was no significant difference in analgesic efficacy between the control group (fentanyl alone) and the experimental group (fentanyl + LA), the experimental group showed significantly reduced adverse reactions and higher levels of patient satisfaction. Duan et al. [92] found that the combination of LA and dexamethasone, a long-acting adrenocortical hormone drug, demonstrated improved analgesic efficacy compared to LA alone, inhibiting tumor growth and reducing nerve compression caused by tumors. Qiu et al. [93] conducted a study on the efficacy of LA patch in managing radiation-induced oral pain among patients with nasopharyngeal carcinoma, showing significantly improved overall effectiveness and reduced adverse reactions. Zhang et al. [94] discussed the efficacy and safety of combining LA with oxycontin for moderate to severe cancer pain. They found that patients receiving the combination therapy required lower doses of oxycontin and experienced a notable decrease in adverse reactions, such as defecation difficulties. Chang et al. [95] examined the efficacy of combining LA with morphine sulfate sustained-release tablets in treating moderate to severe cancer pain among elderly patients. The study demonstrated that different doses of LA effectively enhanced analgesic effects, reduced morphine dosage requirements, and decreased adverse drug reactions. This combination therapy also improved immune function and overall quality of life, with the best outcomes observed in patients receiving 16 mg of LA due to its lower incidence of adverse reactions.

### 5.2. Postoperative Analgesia

Preemptive analgesia has emerged as an efficient method in surgical clinical practice in recent years, demonstrating excellent efficacy in postoperative pain management. Many studies have reported on the clinical utilization of LA for postoperative analgesia [96,97,98,99], highlighting its immense potential for practical implementation.

The studies conducted by Chen et al. [96] and Yang et al. [97] both highlight the potential of LA for postoperative analgesia in orthopedic surgery. Chen et al. [96] found that LA patches exhibit comparable analgesic efficacy to tramadol in orthopedic patients and effectively mitigate postoperative adverse reactions, enhancing patient satisfaction. Yang et al. [97] conducted a study on combining low-dose sufentanil with LA hydrobromide therapy for treating postoperative pain in elderly patients with lower limb fractures, finding that this combination effectively relieves pain and significantly reduces postoperative complications.

Wang et al. [98] extensively investigated the therapeutic effectiveness of LA hydrobromide in relieving postoperative pain among patients undergoing anorectal surgery. The results demonstrated that compared to the control group where patients received no drug analgesia and methadone was only administered for unbearable pain, LA hydrobromide exhibited a higher overall efficacy rate of analgesia, lower postoperative VAS score, and reduced incidence of complications. These findings suggest that LA hydrobromide not only provides effective pain relief but also helps mitigate postoperative complications to some extent in patients undergoing anorectal surgery.

The use of LA for preemptive analgesia after laparoscopic cholecystectomy has also shown excellent clinical efficacy. Wang et al. [99] conducted a comprehensive study on the clinical efficacy of combining LA with parecoxib sodium for preemptive analgesia after laparoscopic gallbladder surgery. The results demonstrated that the combination of parecoxib sodium and LA resulted in significantly lower VAS scores and Ramsay analgesia scores compared to conventional fentanyl anesthesia, along with fewer adverse reactions.

### 5.3. Treatment of Inflammation

The analgesic properties of LA are complemented by its notable anti-inflammatory effects. In 2003, Deng et al. [100] conducted an extensive investigation on the efficacy of LA in a study involving knee osteoarthritis. The findings revealed no statistically significant difference in VAS scores between intra-articular injections of 8 mg LA and those of lidocaine (100 mg) and dexamethasone (3 mg). These results suggest that LA holds promising potential as an anti-inflammatory agent, offering a novel perspective for managing inflammation.

The potential application of LA as an additional treatment for nerve block in scapulohumeral periarthritis was investigated by Li et al. [101]. They conducted suprascapular and axillary nerve blocks using LA on alternate days, followed by local pain point block with LA and rehabilitation training the next day. The results demonstrated a significant reduction in shoulder pain after completing the treatment course, along with notable improvements in the pain point grade and range of motion grade for all sides of the shoulder joint.

Additionally, LA can effectively reduce the inflammatory response in patients and may be used as an adjunct to continuous renal replacement therapy (CRRT) for treating ICU sepsis patients. Yu et al. [102] reported on the clinical efficacy of combining LA with CRRT in ICU patients with sepsis. The control group received conventional CRRT, while the experimental group received CRRT combined with LA. Inflammatory factor levels were recorded before and after treatment, revealing significantly lower levels of TNF-α, hs-CRP, IL-1, and IL-6 in the experimental group compared to the control group.

## 6. Conclusions

The present comprehensive review of advancements in the extraction, biological effects, chemical modification, and clinical application of LA highlights the significant potential of this alkaloid derived from the traditional oriental medicinal plant *Aconitum sinomontanum*. LA has been extensively studied for its diverse pharmacological properties, including analgesic, anti-inflammatory, anti-tumor, anti-arrhythmic, and anti-epileptic activities. Its non-addictive nature and potent efficacy have made it a promising candidate for the management of cancer pain and postoperative analgesia.

The progress in extraction methods and separation techniques has facilitated the efficient isolation and purification of LA, enabling its use in various research and clinical settings. Chemical modifications of LA have further expanded its potential applications by enhancing its solubility, stability, and pharmacological activities. These modifications have also addressed some of the challenges associated with the bioavailability and toxicity of the native compound.

The clinical applications of LA have shown promising results, particularly in the management of pain and inflammation. However, further research is needed to fully understand its mechanisms of action and to optimize its dosing and administration routes. Additionally, the safety and long-term effects of LA need to be thoroughly evaluated to ensure its safe and effective use in clinical practice.

In summary, this review underscores the importance of continued research into LA and its derivatives. Future studies should focus on elucidating the molecular mechanisms underlying its pharmacological effects, exploring new chemical modifications to improve its properties, and conducting large-scale clinical trials to validate its efficacy and safety. Such endeavors will pave the way for the development of novel therapeutic agents based on LA and contribute to the advancement of traditional medicine and modern pharmacology.
